# Routine cancer treatments and their impact on physical function, symptoms of cancer-related fatigue, anxiety, and depression

**DOI:** 10.1007/s00520-021-06787-5

**Published:** 2022-01-11

**Authors:** Niklas Paul Grusdat, Alexander Stäuber, Marion Tolkmitt, Jens Schnabel, Birgit Schubotz, Peter Richard Wright, Henry Schulz

**Affiliations:** 1 Professorship of Sports Medicine/Sports Biology, Chemnitz University of Technology, Thüringer Weg 11, 09126 Chemnitz, Sachsen Germany; 2 Deutsches Rotes Kreuz Krankenhaus (DRK), German Red Cross Hospital, Chemnitz-Rabenstein, Germany; 3 Tumorzentrum Chemnitz e.V., Clinical Cancer Registry, Chemnitz, Germany; 4 Department of Sport, Health Sciences and Social Work, Oxford Brookes University, Oxford, UK

**Keywords:** Physical function, Mental health, Survivorship, Support

## Abstract

**Background and purpose:**

Breast cancer can be a major challenge for affected women. Knowledge of the physical function, symptoms of cancer-related fatigue, anxiety, and depression based on the cancer treatment may help to guide adequate support.

**Methods:**

For this prospective observational study, we collected data from seventy-nine women with a mean age 54.6 ± 9.5 years prior to the onset of breast cancer treatment (T0) and after (T1/T2). Handgrip strength test (HGS), six-minute walk test (6MWT), the phase angle (PhA), the hospital anxiety and depression scale (HADS), and functional assessment of chronic illness therapy-fatigue (FACIT-F) were used to collect data from four treatment subgroups SC, surgery + chemotherapy; SCR, surgery + chemotherapy + radiation therapy; SR, surgery + radiation therapy; and S, surgery.

**Results:**

A mixed ANOVA revealed a significant interaction between time and group for PhA, *F* = 8.55, *p* < 0.01; HGS, *F* = 3.59, *p* < 0.01; 6MWT, *F* = 4.47, *p* < 0.01; and FACIT-F, *F* = 2.77, *p* < 0.05 with most pronounced deterioration seen in group SCR (PhA 4.8°; HGS 27.5 kg, 6MWT 453.4 m, FACIT-F 33.8 points). HADS data displayed moderate anxiety and depression predominantly after treatment.

**Conclusion:**

Our study showed that the extent of change in physical function, symptoms of fatigue, anxiety, and depression depends on the treatment conditions. The potentially higher risk of impaired function due to the prevalence of values below a critical threshold requires early initiated multidisciplinary support.

## Introduction

Breast cancer continues to be the most frequently diagnosed female cancer in the USA [1]. The European Union reported 91,826 cases of death from breast cancer in 2020. About 70,000 new cases are diagnosed in Germany every year. Overall survival has improved in recent decades with new therapy options and personalized medicine [2].

However, breast cancer patients receiving active treatment are often overwhelmed, resulting in various concerns [3]. Despite the underrepresentation in scientific research, social-emotional challenges [4], physical functional limitations [5, 6], experiences of depression [7], debilitating fears [8], and cancer-related fatigue (CRF) [9, 10] have been observed. If unmanaged, deficits may lead to impaired quality of life and the inability to handle instrumental activities of daily living [11, 12]. Especially at an early stage in life, the ability to function in the workplace and employment issues are of great concern [13]. Particularly in stages of cancer I–III, pronounced effects seem not adequately investigated and underestimated in routine clinical assessment. Moreover, a comprehensive patient-orientated picture on prevalence and severity of adverse events at diagnosis, across conventional treatment, and survivorship is lacking.

Handgrip strength (HGS) [14] and the six-minute walk test (6MWT) [15] have gained scientific credibility in the clinical setting as biomarkers of physical function. In addition, the use of bioelectrical impedance analysis (BIA)–derived phase angle (PhA) led to scientific interest because it provides detailed information on body composition, general health [16], and cell membrane integrity [17]. These measures are of prognostic value for an unfavorable clinical outcome, e.g., disease progression [18] and the incidence of postoperative complications [19].

More studies are needed on patients’ perceived circumstances linked to their routine cancer treatment, including chemotherapy, surgery, and radiation therapy. Alongside the traditional clinical reports, transparency on patient-reported outcomes (PROs) is required to enhance the quality of care [20, 21]. Close monitoring patient’s care pathway appears to be of particular relevance as the option to conduct a risk stratification becomes available. Differentiating the patient’s perception of disease and detecting unmet supportive care needs could help personalize and optimize clinical and survivorship care. Few scientific data are available to establish critical threshold values of physical function combined with CRF, anxiety, and depression of women with breast cancer. The purpose of the present study was to determine the extent to which PROs of physical function, CRF, anxiety, and depression change throughout the treatment of breast cancer and in early survivorship.

## Methods

**Fig. 1:**
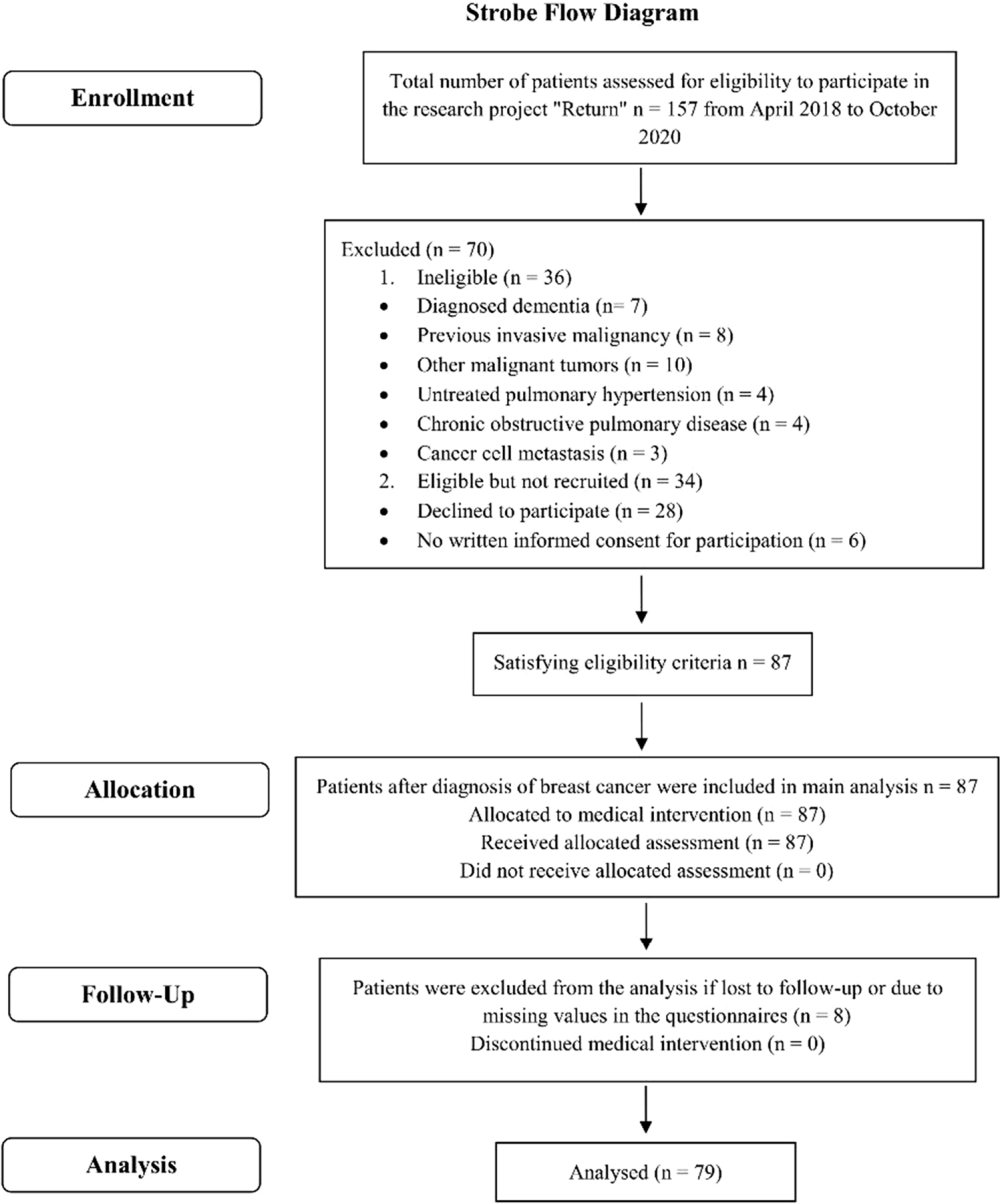
STROBE flow diagram of the prospective observational study in women with breast cancer

Between April 2018 and October 2020, a total of 157 patients with the first diagnosis of breast cancer were recruited within the research study “Return” (trial acronym), which was approved by the ethics committee of Chemnitz University of Technology (V-182–17-AS-Tumor-20012017) and registered with the German Clinical Trials Register (ID: DRKS00014263). Patients were recruited in the Red Cross Hospital in Chemnitz-Rabenstein/Germany. Within 1 week after the breast cancer diagnosis, women were invited by their doctor to consultation and informed about possible participation in the present study. Participants had the opportunity to discuss their participation and consider the information leaflet with detailed information on the research process. Furthermore, sufficient time (> 24 h) to reflect on the implications of participating in the study was given before the patients had to decide. Inclusion criteria for this analysis were patients’ written informed consent, recent diagnosis of untreated female breast cancer, age < 70 years, no defibrillator or cardiac pacemaker, and no orthopedic restrictions for participating in the assessment. Patients were excluded after completing a medical history interview for eligibility if they had a previous invasive malignancy, other malignant tumors, untreated pulmonary hypertension, and chronic obstructive pulmonary disease. Eighty-seven participants who had not initiated cancer treatment met the inclusion criteria and completed the allocated assessments and medical interventions for statistical analysis of this prospective observational study. Eight patients were excluded from the analysis if lost to follow-up or due to missing values in the questionnaires. Further restrictions were recorded as presented in Fig. 1. Data were collected three times, prior to the onset of cancer treatment, at pre-test (T0), and after, at post-test (T1). To be able to keep the schedule, appointments were made immediately and in progress towards completion. The second follow-up (T2) was carried out approximately 3 months after medical treatment for each woman. Cases with long-term endocrine therapy continued beyond T2. Based on the variable duration of breast cancer treatment, repeated testing was performed at different times. Cooperation and coordination between parties involved were required to ensure participation. Four treatment subgroups were included for the following analysis (SC, Surgery + Chemotherapy; SCR, Surgery + Chemotherapy + Radiation Therapy; SR, Surgery + Radiation Therapy; S, Surgery). Baseline demographics, tumor pathology, estrogen receptor, progesterone receptor, and human epidermal growth factor receptor status were provided by the Clinical Cancer Registry Chemnitz (Tumorzentrum Chemnitz) (Table 1).

**Table 1:** Baseline demographics of *n* = 79 women with breast cancer

Variable	Group SC	Group SCR	Group SR	Group S
*N* (%) Age [years]	22 (27.9) 51.9 ± 11.6 (30.0–69.0)	17 (21.5) 54.4 ± 8.5 (41.0–64.0)	27 (34.2) 56.7 ± 9.0 (40.0–69.0)	13 (16.5) 55.3 ± 7.3 (46.0–64.0)
Age, 30–35 years *n* (%) Age, 35–40 years *n* (%) Age, 41–49 years *n* (%) Age, 50–59 years *n* (%) Age, 60–69 years *n* (%) Height [m]	2 (2.5) 2 (2.5) 5 (6.3) 6 (7.6) 7 (8.9) 1.65 ± 0.08 (1.50–1.82)	0 (0.0) 0 (0.0) 6 (7.6) 4 (5.1) 7 (8.9) 1.65 ± 0.08 (1.47–1.78)	0 (0.0) 3 (3.7) 2 (3.7) 9 (11.4) 13 (16.5) 1.61 ± 0.06 (1.45–1.72)	0 (0.0) 0 (0.0) 3 (3.8) 6 (7.6) 4 (5.1) 1.63 ± 0.08 (1.41–1.72)
Weight [kg]	72.1 ± 14.2 (55.7–107.9)	82.7 ± 20.2 (54.1–135.1)	68.6 ± 12.4 (49.3–95.9)	72.6 ± 12.5 (46.9–97.5)
BMI [kg m^−2^]	26.4 ± 5.0 (20.3–38.2)	30.5 ± 6.8 (22.0–45.2)	26.4 ± 4.8 (19.0–37.9)	27.4 ± 4.3 (23.0–37.6)
UICC *n* (%)	IA: 5 (6.3), IIA: 10 (12.7) IIIA: 1 (1.3) IIB: 6 (7.6)	IA: 7 (8.9), IIA:7 (8.9) IB: 2 (2.5) IIB: 1 (1.3)	IA: 25 (31.7), IIA: 2 (2.5) IB: 0 (0.0) IIB: 0 (0.0)	IA: 5 (6.3) IIA: 8 (10.1) IB: 0 (0.0) IIB: 0 (0.0)
Her2/neu status, *n* (%) ER status, *n* (%) MC, *n* (%) IDC, *n* (%) IDC-L, *n* (%) ILC, *n* (%) ICC, *n* (%)	Pos. 1 (1.3) Neg. 21 (26.6) Pos. 13 (16.5) Neg. 9 (11.4) 1 (1.3) 18 (22.8) 0 (0.0) 3 (3.8) 0 (0.0)	Pos. 2 (2.5) Neg. 15 (19.0) Pos. 14 (17.7) Neg. 3 (3.8) 1 (1.3) 16 (20.3) 0 (0.0) 0 (0.0) 0 (0.0)	Pos. 0 (0.0) Neg. 27 (34.2) Pos. 27 (34.2) Neg. 0 (0.0) 0 (0.0) 24 (30.4) 0 (0.0) 2 (2.5) 1 (1.3)	Pos. 13 (16.5) Neg. 0 (0.0) Pos. 13 (16.5) Neg. 0 (0.0) 0 (0.0) 7 (8.9) 1 (1.3) 5 (6.3) 0 (0.0)

Data are expressed as means ± standard deviation (SD); minimum and maximum

*n* = number of patients (percentage)

*SC*, Surgery + Chemotherapy; *SCR*, Surgery + Chemotherapy + Radiation Therapy: SR, Surgery + Radiation Therapy; S, Surgery; *ER*, estrogen receptor; *H HER2/neu*, human epidermal growth factor receptor 2; *ICC*, invasive cribriform carcinoma; *IDC*, invasive ductal carcinoma; *IDC-L*, invasive ductal carcinoma with lobular features; *ILC*, invasive lobular carcinoma; *MC*, mucinous breast carcinoma; *neg*, negative; *pos*, positive; *UICC*, Union for International Cancer Control

### Measurements

Particular patients receiving cytotoxic drugs were reported to have difficulties performing complex assessments. Therefore, in order to address conditions, HGS test with a hydraulic hand dynamometer (Baseline®, HIResTM, Gauge ERTM, USA) was performed. This test requires only a single piece of equipment and minimal effort to conclude muscle mass and muscle function (strength or physical performance) on subjects who may be unwilling or unable to execute other more strenuous tests. In general, it may be a measure of physical fitness as it predicts overall strength and health. Following the Southampton protocol [22], the subject was seated in a standard chair with legs, back support, and fixed arms (the same chair for each measurement), while the feet were placed flat on the floor with the hips and knees positioned at approximately 90°. The participant was instructed to maintain the shoulder slightly abducted (approximately 10°), elbow flexed at 90°, forearm in neutral position (rested on the arms of the chair), thumb facing upwards, and wrist just over the end of the arm of the chair (between 15° and 30° of extension and 0–15° of ulnar deviation). A demonstration showed that gripping very tightly registers the best score. To ensure a comfortable feeling in the hand, the bow handles’ span was adjusted for the individually preferred length. Three trials on each side (alternating) of the right and left hand were performed, starting with the right hand. While holding the dynamometer, the base rested on the palm of the examinators hand. This supported the weight of the dynamometer to negate the effect of gravity on peak strength. Following the protocol, the participant was instructed: “I want you to squeeze as hard as you can for as long as you can until I say stop. Squeeze, squeeze, squeeze, stop” (when the needle stops rising). The maximum grip score (the peak value in kilograms from the outer dial) of all six attempts was used in statistical analyses.

The 6MWT is an evidence-based method to determine treatment effects on cancer patients’ submaximal endurance performance and functional exercise capacity [16]. The valid and reliable measure was used to examine the most excellent possible walking distance on a 20-m (m) track in 6 min [23]. In line with standardization, under no circumstances was the pace increased beyond walking. Walking with other patients or the observer was prohibited for the examination. Prefabricated markings on the floor of the corridor served as route boundaries. An acoustic stop signal was signaling the completion of the test.

The PhA and raw impedance parameters of cell resistance (R) and cell reactance (Xc) were measured using BIA, a non-invasive technique to predict the body composition (BIA® 3 SF, EgoFit GmbH, Germany). After resting for 10 min in supine, the associated bio-signals of R, Xc, and the PhA were recorded on the subject’s right side of the body, between the wrist and ankle via skin electrodes on a non-conductive surface. By applying a harmless, alternating current at a fixed frequency of 50 kHz, a homogeneous electrical energy field was generated by which the conductivity of the human body was measured. The body water and the containing substances were causing a resistive ohmic resistance representing the tissue hydration status of intracellular and extracellular fluid. The electrical charges at the cell membranes were causing a capacitive resistance associated with the nutritional status, the body cell mass, membrane integrity, and skeletal muscle mass. The arctangent between R (pure opposition of a biological conductor) and Xc (capacitative) was computed by using the following equation: $PhA\left[^\circ \right]=\mathrm{arctan}\left(\frac{Xc}{R}\right)\times \left(\frac{180}{\pi }\right)$. The PhA is the essential reference value and an indicator of health [22, 24].

Height and weight were measured with footwear and headwear removed using a standard stadiometer and weigh scale, Seca IEC 601 (Vogel & Halke, Hamburg, Germany). This protocol allows the calculation of the body mass index (BMI). All assessments were performed by personnel trained in densitometry and blinded to the assignment. Mental health is investigated by the hospital anxiety and depression scale (HADS). Fatigue and the impact on daily activities and function were self-reported with the functional assessment of chronic illness therapy-fatigue (FACIT-F). All patients completed the questionnaires with qualified personnel available to answer any questions or clarify the meaning of any of the items.

### Questionnaires

The hospital anxiety (HADS-A) and depression scale (HADS-D) consists of 14 thematically alternately listed questions (points per question: 0–3; total score 0–21). HADS-A and HADS-D are interpreted as follows: 0–7 = normal, 8–10 = borderline abnormal (mild case), 11–14 = abnormal (moderate case), 15–21 = abnormal (severe case). Higher HADS-values represent a more pronounced mental impairment [25]. To determine CRF, the 13-item functional assessment of chronic illness therapy-fatigue (FACIT-F) with a range from 0 to 52 was applied [26]. For FACIT-F, higher scores (negative items were reverse-scored) indicate a non-fatigued status.

### Data analysis

For HGS [27], PhA [28], and 6MWT [29], data can be interpreted and classified in terms of their clinical relevance due to existing critical threshold values in the scientific literature. Critical grip strength is categorized by a value below the individual standardized mean risk threshold of ≥ 1 SD [27]. For the global impedance parameter analysis, the fifth percentile, stratified for sex, age, and BMI, appears as a cutoff and reference for impaired functional status [28]. The sex-specific reference equation $6MWD=2.11 \times {height}_{cm}) - (2.29 \times {weight}_{kg}) - (5.78 \times age) + 667m$ was used to compute and categorize the predicted walking distance (m) for the study individuals [29].

### Statistical analysis

The data analysis was performed with the statistical software package IBM SPSS statistics 26 (Chicago, IL, USA). Only those patients who completed all assessments were included in the analysis. Descriptive statistics are presented as mean, standard deviation (SD), and the minimum and maximum of the outcome parameters. A significance level of *P* < 0.05 was set. To avoid estimation problems, the authors were expecting a moderate to large effect size. The power level of analysis was set at 0.80. Based upon pilot testing, the estimated sample size was sufficient to analyze group effects and significant differences. Demographic characteristics (age, height, weight, BMI) were tested using ANOVA to ensure comparability between the study groups. All metric data were normally distributed (Shapiro–Wilk test). Requirements for applying mixed ANOVA (between-within) were identified in terms of sphericity (Mauchly test) and variances equality (homogeneity) (Levene’s test). A Bonferroni correction was applied to counteract the accumulation of alpha errors and estimation problems, avoiding the likelihood of incorrectly rejecting a null hypothesis (i.e., making a type I error). The main effects for time (entire group and individual groups), the interaction between time and group (difference between groups), and group comparison regardless of the time were tested for significant effects using a mixed ANOVA and post hoc analysis (Tukey, Games-Howell). The primary effect for time on the dependent variables (independently of the group allocation) was investigated with simple main effects of the within-subject factor (Greenhouse–Geisser). The time effect of the individual groups ([mean T0-mean T1, T2]/SD) was tested with a repeated measure analysis of variance for simple main effects of the between-subjects factor (Tukey-HSD) [30]. The effect size was calculated by using the formula $partial {n}^{2}= \frac{{SS}_{effect}}{SSeffect + SSerror}$. Suggested benchmarks for interpretation of the effect size are small (0.1–0.3), medium (0.3–0.5), and large (> 0.5) [31].

## Results

Seventy-nine women with breast cancer were included in the present analysis. The group distribution and patients’ clinical characteristics, and the treatment time are summarized in Table 2. The response rate for patients that had been contacted to participate in this study was 55% (the number of enrollees divided by the number of subjects screened). The mean (SD) age of the total sample at diagnosis was 54.6 ± 9.5 years (range = 30–69 years). The mean (SD) time interval between diagnosis of breast cancer and initial data collection prior to starting treatment for breast cancer (T0) was 6.8 ± 1.3 days (range 6.0–9.0 days). Inclusion cutoff time for consent based on diagnosis was 4.5 ± 1.2 days (range 2.0–7.0 days). For completing treatment, all women with primary disease finished their cycles of chemotherapy, treatment sessions of radiation therapy, and/or cancer surgery. The total mean (SD) time for completing therapy was 6.6 ± 3.0 months (range 1.0–13.4 months). After the end of breast cancer treatment, data were collected within 1 week (mean 5.7 ± 0.8 days, range 4.0–7.0). The second follow-up for each patient took place 3 months after T1 (mean 91.4 ± 1.5 days, range 86.0–97.0).

**Table 2:** Clinical characteristics of *n* = 79 breast cancer patients according to their routine cancer treatment

Variable	Group SC	Group SCR	Group SR	Group S
*n* (%) Time of therapy (month) SNB, *n* (%) ALND, *n* (%) BCS, *n* (%) MRM, *n* (%) SCM, *n* (%) BCS + SCM, *n* (%) ET, *n* (%) Neoadjuvant ET, *n* (%) Adjuvant ET, *n* (%) TMX, *n* (%) ALs, *n* (%) C, *n* (%) Neoadjuvant C, *n* (%) Adjuvant C, *n* (%) E + CP + T + CARB, *n* (%) E + CP + T, *n* (%) DTX + E, *n* (%) E + CP, *n* (%) DTX + CP, *n* (%) T + CARB, *n* (%) DTX + CARB + H, *n* (%) R, *n* (%)	22 (27.9) 7.7 ± 1.3 (6.0–10.5) 21 (26.6) 5 (6.3) 8 (10.1) 2 (2.5) 9 (11.4) 3 (3.8) 14 (17.7) 0 (0.0) 14 (17.7) 1 (1.3) 13 (16.5) 22 (27.8) 10 (12.7) 13 (16.5) 5 (6.3) 7 (8.9) 4 (5.1) 2 (2.5) 1 (1.3) 2 (2.5) 1 (1.3) 0 (0.0)	17 (21.5) 10.4 ± 1.6 (7.1–13.4) 15 (19.0) 3 (3.8) 16 (20.3) 1 (1.3) 0 (0.0) 0 (0.0) 13 (16.5) 1 (1.3) 12 (15.2) 4 (5.1) 9 (11.4) 17 (21.5) 9 (11.4) 8 (10.1) 3 (3.8) 6 (7.6) 5 (6.3) 0 (0.0) 1 (1.3) 0 (0.0) 2 (2.5) 17 (21.5)	27 (34.2) 5.3 ± 1.5 (3.0–9.3) 27 (34.2) 0 (0.0) 27 (34.2) 0 (0.0) 0 (0.0) 0 (0.0) 26 (32.9) 1 (1.3) 25 (31.7) 6 (7.6) 20 (25.3) 0 (0.0) 0 (0.0) 0 (0.0) 0 (0.0) 0 (0.0) 0 (0.0) 0 (0.0) 0 (0.0) 0 (0.0) 0 (0.0) 27 (34.2)	13 (16.5) 2.5 ± 1.6 (1.0–5.6) 13 (16.5) 0 (0.0) 0 (0.0) 2 (2.5) 10 (12.7) 1 (1.3) 12 (15.2) 1 (1.3) 11 (0.0) 1 (1.3) 11 (13.9) 0 (0.0) 0 (0.0) 0 (0.0) 0 (0.0) 0 (0.0) 0 (0.0) 0 (0.0) 0 (0.0) 0 (0.0) 0 (0.0) 0 (0.0)

Data are expressed as means ± standard deviation (SD); min, minimum; max, maximum; *n* = number of patients (percent)

*SC*, Surgery + Chemotherapy; *SCR*, Surgery + Chemotherapy + Radiation Therapy; *SR*, Surgery + Radiation Therapy; *S*, Surgery; *ALND*, axillary lymph node dissection; *ALs*, aromatase inhibitors; *BCS*, breast-conserving surgery; *CARB*, carboplatin; *CP*, cyclophosphamide; *C*, chemotherapy; *DTX*, docetaxel; *E*, epirubicin; *ET*, endocrine therapy; *H*, herceptin (trastuzumab); *MRM*, modified radical mastectomy; *R*, radiation therapy; *SCM*, subcutaneous mastectomy; *SNB*, sentinel node biopsy; *S*, surgery; *T*, paclitaxel; *TMX*, tamoxifen

### Anthropometrics and biomarkers of physical functional status

The statistical comparison of the anthropometric data and the biomarkers of physical functional status is listed in Table 3. The numerical data of the longitudinal comparison indicated a significant main effect for the anthropometric parameters: weight [kg] and BMI [kg/m^2^].

**Table 3:** The primary outcome measures of anthropometrics, physical function, fatigue, anxiety, and depression of the subgroups prior to the onset of (T0), after cancer treatment (T1), and at second follow-up (T2)

Variable	G	Mean (SD)				P^*^–T	*F*–T	*F*–G	*F*–GxT	η^2^ (T)	η^2^ (GxT)
		T0	T1	T2	n						
Weight [kg]	SC SCR SR S	72.1 ± 14.2 82.7 ± 20.24 68.6 ± 12.4 72.6 ± 12.5	69.8 ± 13.7 80.1 ± 21.3 67.4 ± 12.2 71.3 ± 12.3	71.8 ± 13.9 80.4 ± 20.1 67.9 ± 11.7 71.4 ± 12.4	22 17 27 13	a, c a, b a	11.98^***^	2.84^*^	1.18	0.14	0.05
BMI [kg m^−2^]	SC SCR SR S	26.4 ± 5.0 30.5 ± 6.8 26.4 ± 4.8 27.4 ± 4.3	25.6 ± 4.8 29.5 ± 7.0 25.9 ± 4.8 26.9 ± 4.1	26.3 ± 4.8 29.6 ± 6.6 26.1 ± 4.4 26.9 ± 4.2	22 17 27 13	a, c a, b a	12.46^***^	2.23	1.15	0.14	0.04
R 50 kHz [Ohm]	SC SCR SR S	556.4 ± 59.1 514.9 ± 59.7 559.0 ± 47.1 529.5 ± 33.6	582.7 ± 57.8 546.2 ± 60.2 574.9 ± 55.6 532.2 ± 41.2	570.1 ± 55.7 532.7 ± 59.1 566.4 ± 47.0 525.1 ± 41.3	22 17 27 13	a, b, c a, b, c a	37.76^***^	3.35^*^	3.80^**^	0.34	0.13
Xc 50 kHz [Ohm]	SC SCR SR S	55.0 ± 7.2 53.0 ± 7.7 53.0 ± 6.0 51.3 ± 6.8	48.6 ± 7.1 45.9 ± 6.9 47.5 ± 4.9 47.6 ± 6.9	50.4 ± 6.4 46.6 ± 6.6 50.4 ± 5.5 49.5 ± 6.5	22 17 27 13	a, b, c a, b a, b, c a, c	130.96^***^	0.71^NS^	4.51^**^	0.64	0.15
PhA 50 kHz [°]	SC SCR SR S	5.6 ± 0.6 5.9 ± 0.5 5.4 ± 0.6 5.5 ± 0.6	4.8 ± 0.5 4.8 ± 0.5 4.7 ± 0.5 5.1 ± 0.6	5.1 ± 0.5 5.0 ± 0.5 5.1 ± 0.6 5.4 ± 0.6	22 17 27 13	a, b, c a, b a, b, c a, c	188.86^***^	0.78^NS^	8.55^***^	0.72	0.26
HGS peak [kg]	SC SCR SR S	32.7 ± 3.9 32.6 ± 6.0 31.4 ± 3.2 30.8 ± 3.8	27.8 ± 4.5 27.5 ± 5.1 28.4 ± 3.3 28.3 ± 3.5	27.9 ± 4.9 27.2 ± 5.4 28.3 ± 3.1 27.5 ± 3.5	22 17 27 13	a, b a, b a, b a, b	139.06^***^	0.07^NS^	3.59^**^	0.65	0.25
6MWD [m]	SC SCR SR S	514.6 ± 54.3 493.3 ± 56.7 510.3 ± 59.4 529.9 ± 59.9	474.3 ± 60.6 453.4 ± 66.2 487.8 ± 53.0 514.0 ± 61.7	479.1 ± 57.3 460.5 ± 60.6 492.1 ± 51.8 517.6 ± 53.4	22 17 27 13	a, b a, b a, b a, b	99.12^***^	2.11^NS^	4.47^**^	0.57	0.15
FACIT-F (0–52)	SC SCR SR S	45.2 ± 2.8 44.9 ± 2.8 45.1 ± 4.0 45.8 ± 5.2	34.5 ± 2.8 33.8 ± 2.9 36.6 ± 4.9 37.2 ± 4.3	36.2 ± 4.3 35.2 ± 3.9 38.9 ± 5.9 39.9 ± 4.4	22 17 27 13	a, b a, b a, b, c a, b, c	288.87^***^	2.56^NS^	2.77^*^	0.79	0.10
HADS-A (0–21)	SC SCR SR S	8,8 ± 3.4 9.0 ± 2.9 9,2 ± 4.0 8,3 ± 3.7	11,3 ± 3.0 12,6 ± 2.3 10,4 ± 2.8 9,8 ± 2.7	10.8 ± 2.9 12.1 ± 2.7 9.9 ± 2.7 9.2 ± 4.0	22 17 27 13	a, b a, b	18.68^***^	1.92^NS^	1.51^NS^	0.20	0.06
HADS-D (0–21)	SC SCR SR S	6.5 ± 2.9 6.8 ± 3.8 6.9 ± 4.0 7.1 ± 4.6	9.6 ± 3.8 10.5 ± 3.0 9.0 ± 3.3 8.4 ± 3.6	9.6 ± 2.9 10.1 ± 4.1 8.3 ± 3.3 8.1 ± 3.6	22 17 27 13	a, b a, b a a	18.14^***^	0.60^NS^	1.02^NS^	0.20	0.04

Data are expressed as means ± standard deviation (SD); change in percent (%)

*SC*, Surgery + Chemotherapy; *SCR*, Surgery + Chemotherapy + Radiation Therapy; *SR*, Surgery + Radiation Therapy; *S*, Surgery; *n*, number of patients; *NS*, not significant; *T*, time; *G*, group

^*^P < 0.05; ^**^P < 0.01; ^***^P < 0.001

a, T1 differed significantly from baseline; b, T2 differed significantly from baseline; c, T2 differed significantly from T1

Functional status (HGS, 6MWT, PhA) was significantly reduced at T1 and T2, with more significant restrictions experienced in women exposed to chemotherapy.

The mean walking distance of 511.1 m (T0), 481.0 m (T1), and 485.9 m (T2) for the entire group represents low submaximal endurance performance. Considering the age of our study participants (54.6 ± 9.5 years), the overall HGS (T0 = 31.9 kg, T1 = 28.0 kg, T2 = 27.8 kg) indicated a weak muscle strength. Medium to large effects for R, Xc, and PhA were meeting the criteria for abnormal physiology. The most pronounced impact on the state of health at T1 (4.8° ± 0.5) in the SCR group represented a mean reduction of 1.1° from baseline.

### Physical function and the corresponding prevalence of critical values

Substantial deteriorations were recognized post-cancer treatment in SC (91%) and SCR (88%) for a lower limit of the average range [29].

The mean HGS of 32.5 kg was above the risk threshold (26.6 kg) of a large German reference population, including healthy women aged 50–54 years. A detailed view on the study groups pointed out that 41% of women with SCR presented a critically HGS at T1, which decreased to 29% at T2 [27]. Although fewer patients showed values below the risk threshold at T2, a negative trend was detected. This may be explained by a further decline in patients already detected in a state of higher risk for possible future morbidity and mortality. At the same time, others showed a slight improvement in line with the cutoff [27].

Average biomarkers were shown before the onset of cancer treatment. Critical values below the published risk threshold were detected after breast cancer treatment and at second follow-up [28]. In order to examine thresholds at each time point, parameters were displayed in Table 4.

**Table 4:** The prevalence of critical values of bio-impedance phase angle, handgrip strength, and six-minute walk test prior to the onset of (T0), after cancer treatment (T1), and at second follow-up (T2)

Variable	Group	T0	T1	T2
Below risk threshold, PhA, *n* (%)	SC	Yes = 1 (4.5) No = 21 (95.5)	Yes = 11 (50.0) No = 11 (50.0)	Yes = 5 (22.7) No = 17 (77.3)
	SCR	Yes = 0 (0.0) No = 17 (100.0)	Yes = 9 (52.9) No = 8 (47.1)	Yes = 5 (29.4) No = 12 (70.6)
	SR	Yes = 3 (11.1) No = 24 (88.9)	Yes = 13 (48.1) No = 14 (51.9)	Yes = 7 (25.9) No = 20 (74.1)
	S	Yes = 1 (7.7) No = 12 (92.3)	Yes = 3 (23.1) No = 10 (76.9)	Yes = 1 (7.7) No = 12 (92.3)
Below risk threshold, HGS, *n* (%)	SC	Yes = 0 (0.0) No = 22 (100.0)	Yes = 7 (31.8) No = 15 (68.2)	Yes = 6 (27.3) No = 16 (72.7)
	SCR	Yes = 1 (5.9) No = 16 (94.1)	Yes = 7 (41.2) No = 10 (58.8)	Yes = 5 (29.4) No = 12 (70.6)
	SR	Yes = 1 (3.7) No = 26 (96.3)	Yes = 8 (29.6) No = 19 (70.4)	Yes = 7 (25.9) No = 20 (74.1)
	S	Yes = 0 (0.0) No = 13 (100.0)	Yes = 6 (46.2) No = 7 (53.8)	Yes = 5 (38.5) No = 8 (61.5)
Below reference, 6MWT, *n* (%)	SC	Yes = 13 (59.1) No = 9 (40.9)	Yes = 20 (90.9) No = 2 (9.1)	Yes = 17 (77.3) No = 5 (22.7)
	SCR	Yes = 11 (64.7) No = 6 (35.3)	Yes = 15 (88.2) No = 2 (11.8)	Yes = 12 (70.6) No = 5 (29.4)
	SR	Yes = 13 (48.1) No = 14 (51.9)	Yes = 20 (74.1) No = 7 (25.9)	Yes = 15 (55.6) No = 12 (44.4)
	S	Yes = 5 (38.5) No = 8 (61.5)	Yes = 8 (61.5) No = 5 (38.5	Yes = 6 (46.2) No = 7 (53.8)

Data are expressed as *n* = number of patients (percentage) for each group

*SC*, Surgery + Chemotherapy; *SCR*, Surgery + Chemotherapy + Radiation Therapy; *SR*, Surgery + Radiation Therapy; *S*, Surgery

### Cancer-related fatigue

The significant reduction in FACIT-F indicated the presence of experienced fatigue, with more severe conditions based on treatment using cytostatic agents and radiotherapy.

### Anxiety and depression

Abnormal anxieties (moderate case), 38.0% (T0), 62.0% (T1), and 43.0% (T2), and depression (moderate case), 17.7% (T0), 36.7% (T1), and 34.2% (T2), were elucidated for the total group.

## Discussion

Based on the preliminary data of the research study “Return,” we conducted a sub-analysis of the PRO measures of functional and mental status and the state of CRF in women with breast cancer regarding their routine cancer treatment. Clinically established assessment procedures were used prior to the onset of (T0) and post-cancer treatment (T1, T2). Our main findings provide evidence that women with breast cancer showed reduced physical function, mental health, and symptoms of fatigue after breast cancer diagnosis with significant deterioration following treatment. A potentially higher risk of impairment accompanied this due to the prevalence of values below a critical threshold. Across all groups, the most pronounced impact was found in patients with multi-modular conditions.

Women receiving anticancer treatment may get overwhelmed by physical functional changes and emotional challenges. Possibly the PhA can be used as a global marker of health status. Available reference values of healthy adults (*n* = 214,732) across the lifespan (ages 18–70 years) facilitate the interpretation and classification of impedance parameters [28]. According to existing literature, reduced quality of life and impaired functional status are prevalent with risk threshold [28, 32, 33]. In the presented study, the evaluated changes in predicted body composition indicated unfavorable physiology. The decreased body cell mass and increased extracellular mass leading to low PhA may be evidence of clinically relevant malnutrition and functional loss (skeletal muscles) [24]. The valuable global biomarker is positively associated with Xc and negatively associated with R. The significant change in Xc represents the resistive effect produced by the tissue interfaces and cell membranes, suggesting a reduction of membrane function and fewer intact numbers of cells [34]. R as the flow restriction to an electrical current implied a higher water distribution between the extra- and intracellular compartments [35]. The observed state of fluid overload may be due to secondary lymphedema attributed to cancer-specific drug and surgical treatment [36–38]. Women who have lymph node removal followed by radiation therapy have a greater risk of developing swelling (e.g., arms and legs) caused by the congestion of lymph fluid. Taking part in regular physical activity stimulates lymphatic circulation and is therefore recommended [39]. Research on bio-electrical characteristics in connection with physical training initiated early in breast cancer treatment is needed.

The BIA is reliable and easily applicable for an oncology nurse or physiotherapist, as no special training is required. The portable use in various settings and quick feasibility allows long-term monitoring and identification of patients at risk. If accompanied by circadian-related measures, causal inference on insufficient dietary intake, body composition, and daily sleep and activity patterns may be improved [40, 41].

Weak physical performance status and concurrent cancer-related symptoms warrant examination. Limited muscle strength might impair the upper extremities’ functionality and performance of everyday tasks (e.g., the ability to dress, write, or lift small objects) [42, 43]. Findings in patients exposed to anthracycline-taxane-containing chemotherapy could potentially be consistent with peripheral neuropathy and perceived loss of motor function, which can be particularly severe and long-lasting [44]. Besides the potentially life-threatening danger, women with breast cancer face various concerns of possible future challenges (e.g., familial, professional, sexuality, body image, logistical) [45]. Considering associations between the stressful life event and the low physical state raises the potential for a skewed rating affected by motivational and emotional aspects and psychological health [46, 47]. Other underlying mechanisms with possible modifying effects comprise general anesthesia received [48], changes in hormone levels [49], and chronic illness with painful or weak hands [50, 51]. The detected continuous reduced functioning requires close observance beyond the time frame chosen in this study to prevent further decline. Early implementation of routine physical function tests may help health care professionals provide feedback and educated advice about the benefits of physical activity, e.g., resistance training.

Surprisingly, even pre-cancer treatment, retrenchment was detected and may be attributable to certain lifestyle factors, including diet, physical activity, smoking, and alcohol consumption [52]. A healthy individual’s 6MWD ranges from 400 to 700 m (m) and reflects the exercise capacity for daily physical activities [15]. Overall aerobic capacity may indicate the inability to meet job requirements to secure financial stability [53, 54]. The cardiorespiratory fitness of affected women may be enhanced with moderate to vigorous physical activity (MVPA) of at least 150 min per week [52, 55].

The German general population–based FACIT-F norm of women aged 50–59 years with a mean score of 42.6 indicated the presence of fatigue in our study population [56]. Intense fatigue (e.g., “I have to limit my social activity because I am tired”) was experienced by most women post-treatment. Severity is shaped by more significant pain, sleep disruption, distress, lower activity, and lower physical and social health status [55]. Furthermore, the proposed CRF is marked by increased lifestyle stresses, such as lack of ability to work and child or elderly care, and potentiated by the treatment time and younger age. For a holistic approach to reduce symptoms, a multidisciplinary support intervention program should contain pain therapy [57], nutritional medicine [58], and exercise therapy [59, 60].

Mental impairment of anxiety and depression was linked to early stages of cancer (stages I–III). Especially, combination therapy evoked restlessness and tension with moderate to severe expression. It is difficult to distinguish between a timely fearful reaction and an anxiety symptom that requires intervention. Therapy appears to be necessary if the behavior and experience of the patients’ everyday lives are impaired. Often, anxiety disorders or depression are not recognized or dismissed as an understandable reaction to a life-threatening illness. Promoting social support, particularly emotional support from family, may positively impact psychological stress and psychiatric morbidity [61]. For more clarity, routine assessments of psychiatric morbidities need to achieve widespread implementation in oncological care. By identifying those patients who require psycho-social support, therapeutic outcomes may be improved. A more substantial alignment with worries about the deterioration of conditions is favored, while the attitude of helplessness or hopelessness is associated with poorer breast cancer prognosis [62]. Women need to receive information about the adverse effects of cancer treatment and advice about coping methods [63, 64].

The present study adds to the existing literature on patient experiences of cancer care. A clear benefit was that patient-orientated indicators and critical points of interest could be assessed quickly and easily with accepted measures. By carefully comprehending patients’ treatment conditions, prospective capturing of perceived circumstances may be improved. Study designs must employ baseline testing to detect changes accurately. PROs may give health care professionals and the multidisciplinary team involved, including oncologists, physiotherapists, and nurses, guidance to determine individualized needs. Practical support in subsequent oncological rehabilitation treatment requires the most appropriate modalities and timing for initiation.

### Limitations

There are limitations to the present study as we could not include an additional follow-up analysis. Since the number of patients, especially in group S, was small (*n* = 13), our findings can only be regarded as preliminary, and future investigations are necessary for the generalizability of our findings. Treatment groups may not represent all cancer patients and especially not for those with severe course of illness. Socioeconomic status as a possible influencing factor was not investigated. Screening patients for their physical activity levels may have led to a more differentiated assumption of study results.

## Conclusion

In summary, women with breast cancer showed decreased physical function, mental health, and symptoms of fatigue. Across all groups, the most pronounced impact was found in patients with multi-modular conditions. A potentially higher risk of impaired function accompanied this due to the prevalence of values below a critical threshold. Group differences were particularly noticeable in the reduced HGS, 6MWT, PhA, mental status, and in the heightened state of fatigue. Based on our findings, multidisciplinary support initiated early in breast cancer treatment seems appropriate to address conditions. The permanent adoption of PROs in clinical research increases the transparency of patients’ perceived circumstances. Routine assessment may lead to an individual risk stratification, which could help personalize and optimize clinical and survivorship care.

## Data Availability

The datasets used and/or analyzed during the current study are available from the corresponding author on reasonable request.

## Code availability

N/A.

## References

[1] Siegel RL, Miller KD, Fuchs HE (2021). Jemal A (2021) Cancer statistics. CA Cancer J Clin.

[2] Barnes B, Kraywinkel K, Nowossadeck E, Schönfeld I, Starker A, Wienecke A, Wolf U (2016) Bericht zum Krebsgeschehen in Deutschland 2016. Robert Koch-Institut. 10.17886/rkipubl-2016-014

[3] Corey B, Smania MA, Spotts H, Andersen M (2020). Young women with breast cancer: treatment, care, and nursing implications. Clin J Oncol Nurs.

[4] Thorn DR, Ladewig Hess AR (2021). Outpatient breast cancer treatment after the hospital: what’s next?-adjuvant medical therapies, management of side effects and common fears, planing and coordination of optimal follow-up care in view of current guidelines. Therapeutische Umschau Revue Therapeutique.

[5] Kubo Y, Naito T, Mori K, Osawa G, Aruga E (2017). Skeletal muscle loss and prognosis of breast cancer patients. Support Care Cancer.

[6] Ten Tusscher M, Groen W, Geleijn E, Sonke G, Konings I, Van der Vorst M, van Zweeden A, Aaronson N, Stuiver MM (2019). Physical problems, functional limitations, and preferences for physical therapist-guided exercise programs among Dutch patients with metastatic breast cancer: a mixed methods study. Support Care Cancer.

[7] Härtl K, Engel J, Herschbach P, Reinecker H, Sommer H, Friese K (2010) Personality traits and psychosocial stress: quality of life over 2 years following breast cancer diagnosis and psychological impact factors. Psycho-Oncology: J Psychol Soc Behav Dimens Cancer 19(2):160–169 10.1002/pon.1536 19189279

[8] Koch L, Bertram H, Eberle A, Holleczek B, Schmid-Höpfner S, Waldmann A, Zeissig SR, Brenner H, Arndt V (2014). Fear of recurrence in long-term breast cancer survivors—still an issue. Results on prevalence, determinants, and the association with quality of life and depression from the Cancer Survivorship—a multi-regional population-based study. Psycho-Oncology.

[9] Bower JE, Wiley J, Petersen L, Irwin MR, Cole SW, Ganz PA (2018). Fatigue after breast cancer treatment: biobehavioral predictors of fatigue trajectories. Health Psychol.

[10] Bower JE (2014). Cancer-related fatigue—mechanisms, risk factors, and treatments. Nat Rev Clin Oncol.

[11] Gold M, Dunn LB, Phoenix B, Paul SM, Hamolsky D, Levine JD, Miaskowski C (2016). Co-occurrence of anxiety and depressive symptoms following breast cancer surgery and its impact on quality of life. Eur J Oncol Nurs.

[12] Montemurro F, Mittica G, Cagnazzo C, Longo V, Berchialla P, Solinas G, Culotta P, Martinello R, Foresto M, Gallizioli S (2016). Self-evaluation of adjuvant chemotherapy-related adverse effects by patients with breast cancer. JAMA Oncol.

[13] Ahn E, Cho J, Shin DW, Park BW, Ahn SH, Noh D-Y, Nam SJ, Lee ES, Yun YH (2009). Impact of breast cancer diagnosis and treatment on work-related life and factors affecting them. Breast Cancer Res Treat.

[14] Kilgour R, Vigano A, Trutschnigg B, Lucar E, Borod M, Morais J (2013). Handgrip strength predicts survival and is associated with markers of clinical and functional outcomes in advanced cancer patients. Support Care Cancer.

[15] Enright PL (2003). The six-minute walk test. Respir Care.

[16] Piccoli A, Rossi B, Pillon L, Bucciante G (1994). A new method for monitoring body fluid variation by bioimpedance analysis: the RXc graph. Kidney Int.

[17] Kumar S, Dutt A, Hemraj S, Bhat S, Manipadybhima B (2012). Phase angle measurement in healthy human subjects through bio-impedance analysis. Iran J Basic Med Sci.

[18] Norman K, Stobäus N, Zocher D, Bosy-Westphal A, Szramek A, Scheufele R, Smoliner C, Pirlich M (2010). Cutoff percentiles of bioelectrical phase angle predict functionality, quality of life, and mortality in patients with cancer. Am J Clin Nutr.

[19] Norman K, Stobäus N, Pirlich M, Bosy-Westphal A (2012). Bioelectrical phase angle and impedance vector analysis–clinical relevance and applicability of impedance parameters. Clin Nutr.

[20] Pappot H, Baeksted CW, Nissen A, Knoop A, Mitchell SA, Christensen J, Hjollund NH, Johansen C (2021). Clinical effects of assessing electronic patient-reported outcomes monitoring symptomatic toxicities during breast cancer therapy: a nationwide and population-based study. Breast Cancer.

[21] Kowalski C, Graeven U, von Kalle C, Lang H, Beckmann MW, Blohmer J-U, Burchardt M, Ehrenfeld M, Fichtner J, Grabbe S (2017). Shifting cancer care towards multidisciplinarity: the cancer center certification program of the German cancer society. BMC Cancer.

[22] Malecka-Massalska T, Chara K, Smolen A, Kurylcio A, Polkowski W, Lupa-Zatwarnicka K (2012). Bioimpedance vector pattern in women with breast cancer detected by bioelectric impedance vector analysis. Preliminary observations. Ann Agric Environ Med.

[23] Schmidt K, Vogt L, Thiel C, Jäger E, Banzer W (2013). Validity of the six-minute walk test in cancer patients. Int J Sports Med.

[24] Marroni CA, Miranda D, Boemeke L, Fernandes SA (2017) Phase angle bioelectrical impedance analysis (BIA) as a biomarker tool for liver disease. Biomarkers in Liver Disease (Biomarkers in Disease: Methods, Discoveries and Applications) Berlim: Springer Science:735–751

[25] Stern AF (2014). The hospital anxiety and depression scale. Occup Med.

[26] Yellen SB, Cella DF, Webster K, Blendowski C, Kaplan E (1997). Measuring fatigue and other anemia-related symptoms with the functional assessment of cancer therapy (FACT) measurement system. J Pain Symptom Manage.

[27] Steiber N (2016). Strong or weak handgrip? Normative reference values for the German population across the life course stratified by sex, age, and body height. PloS one.

[28] Bosy-Westphal A, Danielzik S, Dörhöfer RP, Later W, Wiese S, Müller MJ (2006). Phase angle from bioelectrical impedance analysis: population reference values by age, sex, and body mass index. J Parenter Enter Nutr.

[29] Enright PL, Sherrill DL (1998). Reference equations for the six-minute walk in healthy adults. Am J Respir Crit Care Med.

[30] Field A (2009). Discopering statistics using SPSS.

[31] Bakeman R (2005). Recommended effect size statistics for repeated measures designs. Behav Res Methods.

[32] Norman K, Wirth R, Neubauer M, Eckardt R, Stobäus N (2015). The bioimpedance phase angle predicts low muscle strength, impaired quality of life, and increased mortality in old patients with cancer. J Am Med Dir Assoc.

[33] Lee SY, Lee YJ, Yang J-H, Kim C-M, Choi W-S (2014). The association between phase angle of bioelectrical impedance analysis and survival time in advanced cancer patients: preliminary study. Korean J Fam Med.

[34] Barbosa-Silva MCG, Barros AJ (2005). Bioelectrical impedance analysis in clinical practice: a new perspective on its use beyond body composition equations. Curr Opin Clin Nutr Metab Care.

[35] Buffa R, Mereu R, Putzu P, Floris G, Marini E (2010). Bioelectrical impedance vector analysis detects low body cell mass and dehydration in patients with Alzheimer’s disease. J Nutr Health Aging.

[36] Choi S-M, Lee S-H, Yang Y-S, Kim B-C, Kim M-K, Cho K-H (2001). 5-fluorouracil-induced leukoencephalopathy breast cancer. J Korean Med Sci.

[37] Cormier JN, Askew RL, Mungovan KS, Xing Y, Ross MI, Armer JM (2010). Lymphedema beyond breast cancer: a systematic review and meta-analysis of cancer-related secondary lymphedema. Cancer.

[38] Feliciano EMC, Chen WY, Lee V, Albers KB, Prado CM, Alexeeff S, Xiao J, Shachar SS, Caan BJ (2020). Body composition, adherence to anthracycline and taxane-based chemotherapy, and survival after nonmetastatic breast cancer. JAMA Oncol.

[39] Johansson K, Karlsson K, Nikolaidis P (2015). Evidence-based or traditional treatment of cancer-related lymphedema. Lymphology.

[40] Taetzsch A, Roberts SB, Bukhari A, Lichtenstein AH, Gilhooly CH, Martin E, Krauss AJ, Hatch-McChesney A, Das SK (2021). Eating timing: associations with dietary intake and metabolic health. J Acad Nutr Diet.

[41] Berger AM, Wielgus K, Hertzog M, Fischer P, Farr L (2010). Patterns of circadian activity rhythms and their relationships with fatigue and anxiety/depression in women treated with breast cancer adjuvant chemotherapy. Support Care Cancer.

[42] Celis-Morales CA, Welsh P, Lyall DM, Steell L, Petermann F, Anderson J, Iliodromiti S, Sillars A, Graham N, Mackay DF (2018) Associations of grip strength with cardiovascular, respiratory, and cancer outcomes and all cause mortality: prospective cohort study of half a million UK Biobank participants. Bmj 361 10.1136/bmj.k1651 PMC5939721 29739772

[43] Wu Y, Wang W, Liu T, Zhang D (2017). Association of grip strength with risk of all-cause mortality, cardiovascular diseases, and cancer in community-dwelling populations: a meta-analysis of prospective cohort studies. J Am Med Dir Assoc.

[44] Miltenburg N, Boogerd W (2014). Chemotherapy-induced neuropathy: a comprehensive survey. Cancer Treat Rev.

[45] Corey B, Smania MA, Spotts H, Andersen M (2020). Young women with breast cancer: treatment, care, and nursing implications. Clin J Oncol Nurs.

[46] Cairney J, Dudley D, Kwan M, Bulten R, Kriellaars D (2019). Physical literacy, physical activity and health: toward an evidence-informed conceptual model. Sports Med.

[47] Brown DM, Graham JD, Innes KI, Harris S, Flemington A, Bray SR (2020). Effects of prior cognitive exertion on physical performance: a systematic review and meta-analysis. Sports Med.

[48] Pei D-Q, Zhou H-M, Zhou Q-H (2019). Grip strength can be used to evaluate postoperative residual neuromuscular block recovery in patients undergoing general anesthesia. Medicine.

[49] Boing L, Vieira MdCS, Moratelli J, Bergmann A, de Azevedo Guimarães AC (2020) Effects of exercise on physical outcomes of breast cancer survivors receiving hormone therapy–a systematic review and meta-analysis. Maturitas 10.1016/j.maturitas.2020.06.022 33036706

[50] Cantarero-Villanueva I, Fernandez-Lao C, Fernández-DE-Las-Peñas C, Díaz-Rodríguez L, Sanchez-Cantalejo E, Arroyo-Morales M (2011). Associations among musculoskeletal impairments, depression, body image and fatigue in breast cancer survivors within the first year after treatment. Eur J Cancer Care.

[51] Bohannon RW (2015). Muscle strength: clinical and prognostic value of hand-grip dynamometry. Curr Opin Clin Nutr Metab Care.

[52] Santa Mina D, Brahmbhatt P, Lopez C, Baima J, Gillis C, Trachtenberg L, Silver JK (2017). The case for prehabilitation prior to breast cancer treatment. PM&R.

[53] Kehmeier ES, Sommer MH, Galonska A, Zeus T, Verde P, Kelm M (2016). Diagnostic value of the six-minute walk test (6MWT) in grown-up congenital heart disease (GUCH): Comparison with clinical status and functional exercise capacity. Int J Cardiol.

[54] Islam T, Dahlui M, AbdMajid H, Nahar AM, Taib NAM, Su TT (2014). Factors associated with return to work of breast cancer survivors: a systematic review. BMC Public Health.

[55] Scott JM, Thomas SM, Peppercorn JM, Herndon JE, Douglas PS, Khouri MG, Dang CT, Yu AF, Catalina D, Ciolino C (2020). Effects of exercise therapy dosing schedule on impaired cardiorespiratory fitness in patients with primary breast cancer: a randomized controlled trial. Circulation.

[56] Montan I, Löwe B, Cella D, Mehnert A, Hinz A (2018). General population norms for the functional assessment of chronic illness therapy (FACIT)-fatigue scale. Value Health.

[57] Abrahams H, Gielissen M, Verhagen C, Knoop H (2018). The relationship of fatigue in breast cancer survivors with quality of life and factors to address in psychological interventions: a systematic review. Clin Psychol Rev.

[58] Pereira PTVT, Reis AD, Diniz RR, Lima FA, Leite RD, da Silva MCP, Guerra RNM, de Moraes Vieira ÉB, Garcia JBS (2018). Dietary supplements and fatigue in patients with breast cancer: a systematic review. Breast Cancer Res Treat.

[59] Dong B, Xie C, Jing X, Lin L, Tian L (2019). Yoga has a solid effect on cancer-related fatigue in patients with breast cancer: a meta-analysis. Breast Cancer Res Treat.

[60] Juvet L, Thune I, Elvsaas IØ, Fors E, Lundgren S, Bertheussen G, Leivseth G, Oldervoll L (2017). The effect of exercise on fatigue and physical functioning in breast cancer patients during and after treatment and at 6 months follow-up: a meta-analysis. Breast.

[61] Lueboonthavatchai P (2007). Prevalence and psychosocial factors of anxiety and depression in breast cancer patients. J Med Assoc Thai.

[62] Watson M, Homewood J, Haviland J, Bliss JM (2005). Influence of psychological response on breast cancer survival: 10-year follow-up of a population-based cohort. Eur J Cancer.

[63] Munir F, Kalawsky K, Lawrence C, Yarker J, Haslam C, Ahmed S (2011). Cognitive intervention for breast cancer patients undergoing adjuvant chemotherapy: a needs analysis. Cancer Nurs.

[64] Hashemi S-M, Balouchi A, Al-Mawali A, Rafiemanesh H, Rezaie-Keikhaie K, Bouya S, Dehghan B, Farahani MA (2019). Health-related quality of life of breast cancer patients in the Eastern Mediterranean region: a systematic review and meta-analysis. Breast Cancer Res Treat.

